# Supplementary material for: DMRforPairs: identifying Differentially Methylated Regions between unique samples using array based methylation profiles
Source: BMC Bioinformatics. 2014 May 15;15:141. doi: 10.1186/1471-2105-15-141 (PMC4046028; doi:10.1186/1471-2105-15-141)
Supplement: Additional file 2 — DMRforPairs output for the comparison of A431-MCF7 and NA17018-NA17105. Please start from the HTML files in each folder. Available via the BMC Bioinformatics website. [file 1471-2105-15-141-S2.zip › 1394847754114233_MOESM2_ESM/A431_MCF7/figures/12302.pdf]

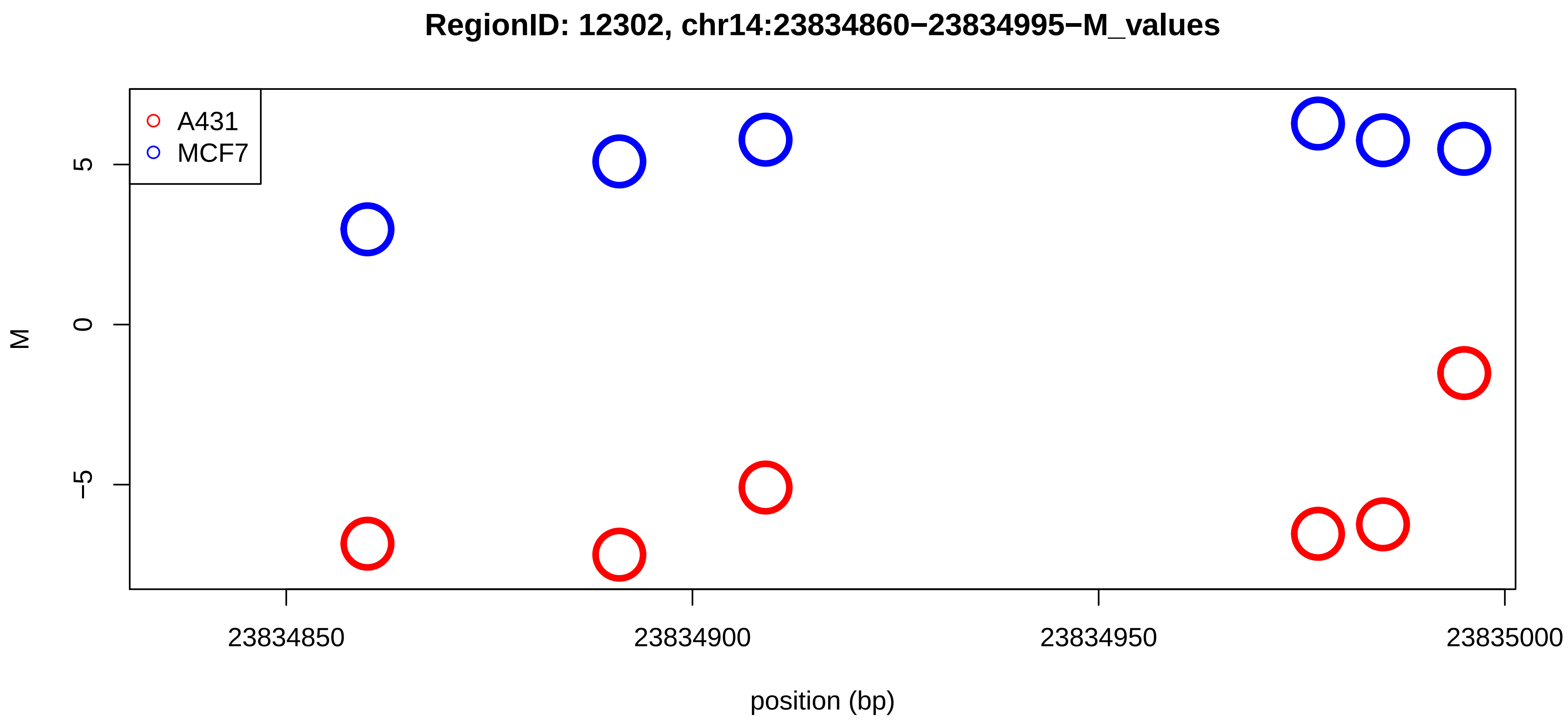

RegionID: 12302, chr14:23834860−23834995−M_values
A431
MCF7
M
5
0
−5
23834850
23834900
23834950
23835000
position (bp)

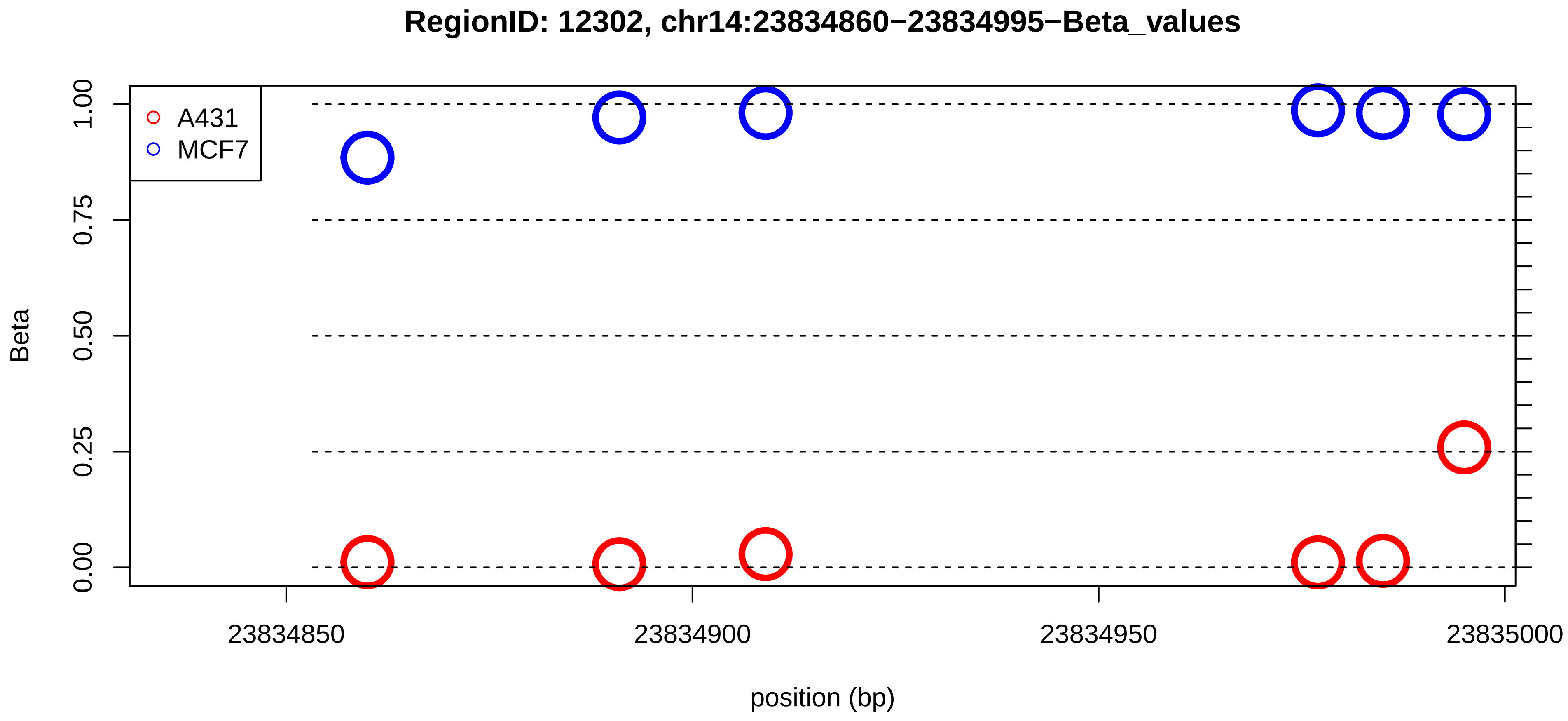

RegionID: 12302, chr14:23834860−23834995−Beta_values
A431
MCF7
Beta
1.00
0.75
0.50
0.25
0.00
23834850
23834900
23834950
23835000
position (bp)

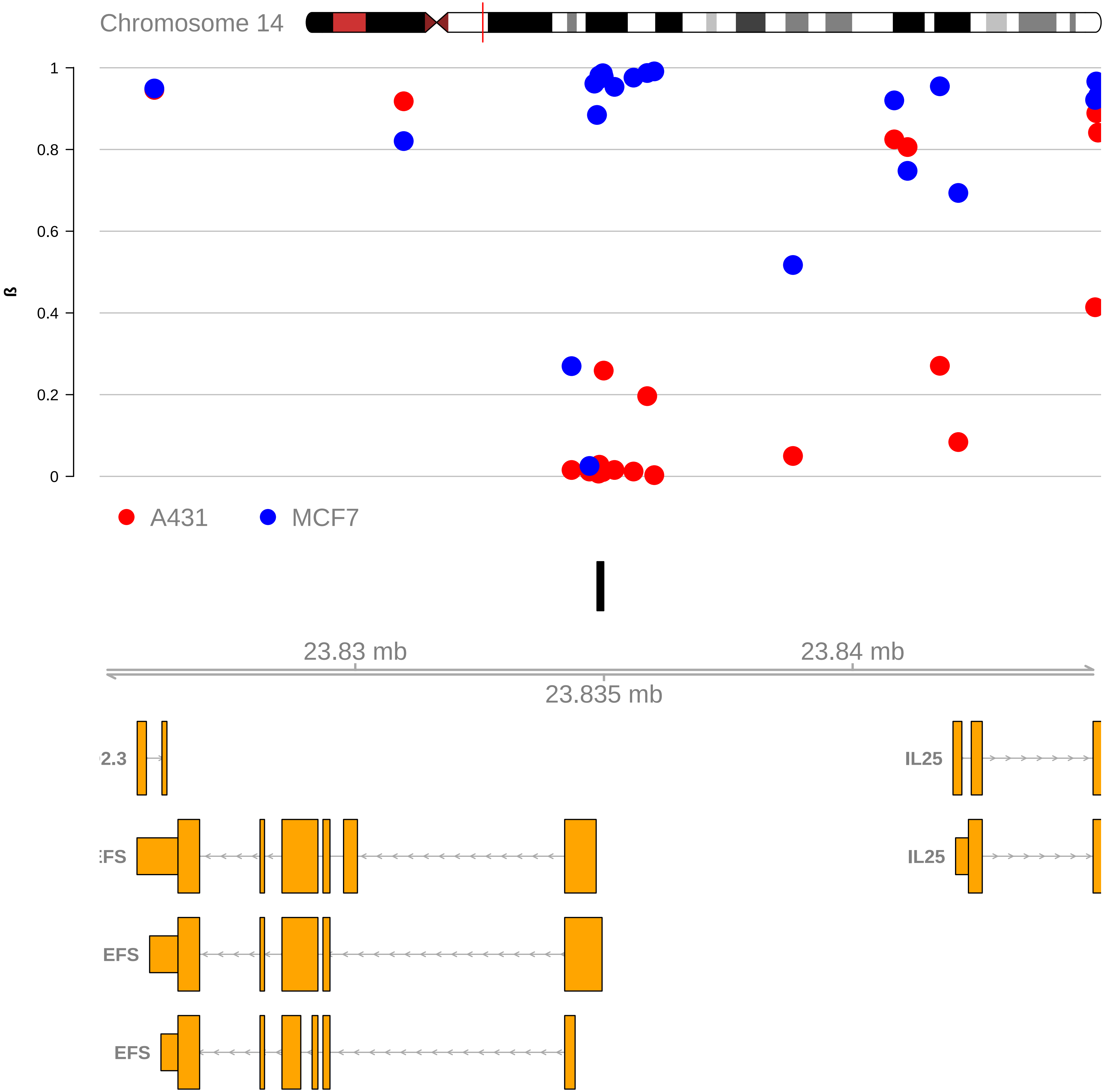

Chromosome 14
1
0.8
0.6
0.4
0.2
0
ß
A431
MCF7
23.83 mb
23.84 mb
23.835 mb
2.3
IL25
EFS
IL25
EFS
EFS
